# Highlighting *Astyanax* Species Diversity through DNA Barcoding

**DOI:** 10.1371/journal.pone.0167203

**Published:** 2016-12-19

**Authors:** Bruno César Rossini, Carlos Alexandre Miranda Oliveira, Filipe Augusto Gonçalves de Melo, Vinicius de Araújo Bertaco, Juan M. Díaz de Astarloa, Juan J. Rosso, Fausto Foresti, Claudio Oliveira

**Affiliations:** 1 Laboratório de Biologia e Genética de Peixes, Departamento de Morfologia, UNESP—Universidade Estadual Paulista, Botucatu, São Paulo, Brazil; 2 Instituto de Biotecnologia da UNESP, UNESP—Universidade Estadual Paulista, Botucatu, São Paulo, Brazil; 3 Universidade Estadual de Maringá, Programa de Pós-Graduação em Ecologia de Ambientes Aquáticos Continentais, Maringá, Paraná, Brazil; 4 Universidade Estadual do Piauí, Campus Alexandre Alves de Oliveira, Parnaíba, Piauí, Brazil; 5 Museu de Ciências Naturais, Fundação Zoobotânica do Rio Grande do Sul, Porto Alegre, Rio Grande do Sul, Brazil; 6 Grupo de Biotaxonomía Morfológica y Molecular de Peces, Instituto de Investigaciones Marinas y Costeras, Universidad Nacional de Mar del Plata. Consejo Nacional de Investigaciones Científicas y Técnicas (CONICET), Buenos Aires, Argentina; Centre National de la Recherche Scientifique, FRANCE

## Abstract

DNA barcoding has been used extensively to solve taxonomic questions and identify new species. Neotropical fishes are found in a wide variety of shapes and sizes, with a large number of species yet to be described, many of which are very difficult to identify. Characidae is the most species-rich family of the Characiformes, and many of its genera are affected by taxonomic uncertainties, including the widely-distributed, species-rich genus *Astyanax*. In this study, we present an extensive analysis of *Astyanax* covering almost its entire area of occurrence, based on DNA barcoding. The use of different approaches (ABGD, GMYC and BIN) to the clustering of the sequences revealed ample consistency in the results obtained by the initial cutoff value of 2% divergence for putative species in the Neighbor-Joining analysis using the Kimura-2-parameter model. The results indicate the existence of five *Astyanax* lineages. Some groups, such as that composed by the trans-Andean forms, are mostly composed of well-defined species, and in others a number of nominal species are clustered together, hampering the delimitation of species, which in many cases proved impossible. The results confirm the extreme complexity of the systematics of the genus *Astyanax* and show that DNA barcoding can be an useful tool to address these complexes questions.

## Introduction

Enormously diverse, Neotropical freshwater fish can be found from South America to southern North America, with more than 7000 recognized species [1] representing 71 families [2] mainly arranged in two major groups, the Characiformes and the Siluriformes [3]. The characiforms are the most diverse group, with a huge variety of body shapes and sizes, found in the lakes and rivers of the Neotropical region, as well as Africa [4]. The most diverse family of this order is the Characidae, which recently reached a total of more than 1100 recognized species [3].

Given its species diversity, the Characidae is also the Neotropical fish family with the most taxonomic problems [5]. The relationships within the family are largely uncertain and many genera are not monophyletic, including *Astyanax*, which is the one of the most species-rich characid genus [5, 6]. Eigenmann [7, 8] presented the first substantial review of the genus, recognizing 74 species and subspecies, many of which have now been re-assigned to other genera, such as *Jupiaba* Zanata, 1997. The second major review of the twentieth century, Géry [4], differed little from Eigenmann’s original proposal. By 2003, the genus had 86 valid species [9], although this number has now increased to 147, of which, approximately 20% were described in the past 10 years [3].

Many *Astyanax* species are currently identified at genus level [10]. A number of authors have also proposed the existence of species complexes, such as those of *Astyanax bimaculatus* Linnaeus, 1758 (the *A*. *bimaculatus* species complex) [11, 12], *Astyanax fasciatus* Cuvier, 1819 (the *A*. *fasciatus* species complex) [13] and *Astyanax scabripinnis* Jennys, 1842 (the *A*. *scabripinnis* species complex) [14]. These groups, together with the large number of other *Astyanax* species, reflect the highly complex nature of the identification of Neotropical characiform species, due to the phenotypic plasticity of the morphological characters traditionally used for species determination, resulting in many identification errors.

One potential solution for these taxonomic problems is the use of molecular species identification. In this context, DNA barcoding has been extensively and successfully used for fish species identification and the resolution of many taxonomic problems. The first study of marine fishes, for example, obtained a 100% success rate in species identification with no overlap found [15], and a recent study also identified correctly almost all the species in a study of the coastal fish fauna in India [16]. Success rates have been lower for freshwater groups, however, with a correct identification rate of 93% being recorded in Mexico and Guatemala [17] and Canada [18], although another study did obtain a success rate of 99.2% in the upper Paraná Basin in Brazil [19].

More than a decade after the molecular identification system was first proposed [20], a variety of different cutoff values for species based on genetic distances have been tested. While some use a cutoff of 1%, for example [21], values of 2% and 3% are most common in studies of Neotropical fish [19, 22–26]. Other criteria include one order of magnitude (10 x) greater than the mean intraspecific divergence [27] and the barcoding gap [28]. More recently, new methods have been proposed for the automatic species identification, such as the Automatic Barcode Gap Discovery (ABGD), the Barcode Index Number (BIN) and the Generalized Mixed Yule Coalescent model (GMYC). The ABGD is an automatic identification procedure that forms clusters of sequences of possible species, based on the distances and differences between intra- and interspecific levels of variation, detecting boundaries even when the distribution overlaps [29, 30]. The BIN is also based on distance methods, clustering sequences with a 2.2% threshold, followed by a Markov analysis [31]. Other approaches are not based only on distance as the criterion for species discrimination [32–34]. The GMYC analysis uses an ultrametric tree to establish species limits, based on a mixture of the Yule (pure-birth) [35] and Kingman models (coalescence) [36], where the algorithm computes the probability of splits in a lineage based on speciation rates, thus identifying a cutoff value which enables the identification of the point at which species or populations split [37].

Given the considerable difficulties for the identification of *Astyanax* species based on morphological traits, and the potential existence of species complexes, the present study investigated the genetic diversity of the genus based on a DNA barcoding approach. With this, we hope to expand our knowledge of one of the most species diverse Neotropical fish genera.

## Material and Methods

### Ethical statement

We declare that the fish under study are not protected under wildlife conservation, and no experimentation was conducted on live specimens. All specimens used were collected in accordance local laws, and in Brazil the sampling was approved by the Brazilian Institute of Environment and Renewable Natural Resources (IBAMA) and Sistema de Autorização e Informação em Biodiversidade (SISBIO) under a license issued in the name of Dr. Claudio Oliveira (SISBIO number 13843–1). After collection, the animals were anesthetized and sacrificed using 1% benzocaine in water as approved by the Bioscience Institute/UNESP Ethics Committee on the Use of Animals (CEUA; protocol 405) and recommended by the National Council for the Control of Animal Experimentation and the Federal Board of Veterinary Medicine.

### Specimen collection

*Astyanax* specimens, a total of 1309 fishes, were collected in a number of different river basins in Argentina, Brazil, Colombia, Guyana, Peru and Venezuela (S1 Table). Additionally, 366 samples were obtained from GenBank. For *Astyanax* sp. we followed the previously existing identification system from Ornelas-Garcia et al. (2008; *Astyanax* sp. 1 to 9 from Mesoamerica). All the other *Astyanax* sp. are named in sequential order from this study. Tissue samples from Argentina were provided by the fish collection of the Coastal and Marine Research Institute (IIMyC) at Universidad Nacional de Mar del Plata in Mar del Plata, Argentina. The tissue samples used for the molecular analyses were preserved in absolute ethanol and stored at -20°C. The voucher specimens were fixed in a 10% formalin solution and are preserved in 70% ethanol. The morphological vouchers were deposited in the fish collection of the Fish Biology and Genetics Laboratory (LBP) at Paulista State University (Universidade Estadual Paulista) in Botucatu, Brazil or Universidad Nacional de Mar del Plata, Argentina. Species identification was based on morphological traits as meristic and morphometric data, color pattern and teeth morphology which are arranged or presented on dichotomic keys, original descriptions, redescriptions and taxonomic reviews [4, 7, 11–13, 38–64]. Consensus sequences were deposited in the BOLD database in the dataset named "BAST- Barcoding *Astyanax*".

### DNA extraction, PCR amplification and sequencing

Total DNA was extracted from muscle fragments following the protocol of the Canadian Center for DNA-Barcoding (CCDB), available at http://www.ccdb.ca. A segment of the 5' region of the mitochondrial COI gene was amplified using different combinations of primers, including L5698-Asn [65], FishF1, FishF2, FishR1 and FishR2 [15], C_FishF1t1–C_FishR1t1 cocktail [66], and H7271-COI [67]. Polymerase chain reactions (PCR) were run in a 12.5 μl volume containing: 1 μl DNA (concentration 50 ng/μl), 0.25 μl each of the forward and reverse primers (concentration 10 mM), 1.25 μl of reaction buffer, 0.2 μl of 200 mM dNTPs mix, 0.37 μl of MgCl_2_ and 0.0625 μl (5 units/μl) of Platinum Taq DNA polymerase (Invitrogen).

The samples were amplified in a Veriti^®^ 96-well thermocycler (Applied Biosystems), with initial denaturation of 5 minutes at 96°C followed by 35 cycles at 96°C for 45 seconds, 54°C for 45 seconds, 72°C for 1 minute, and final extension at 72°C for 1 minute. The amplified PCR products were cleaned up with ExoSAP-IT (USB Corporation) and sequenced in both directions using the BigDye Terminator v3.1 Cycle Sequencing kit (Life Technologies) in an ABI3130 Genetic Analyzer automated sequencer (Applied Biosystems).

### Data analysis

The sequences were edited in BioEdit 7.0.9.0 [68] and aligned in MUSCLE (Multiple Sequence Comparison by Log-Expectation) [69]. The first analysis were conduct based on genetic distances calculated in MEGA 5 [70], using the Kimura-2-parameter (K2P) substitution model [71] to estimate the Neighbor-Joining (NJ) tree [72], based on a 2% cutoff value. This cutoff was used to define the initial clusters and then we tested all the NJ clusters defined by the 2% cutoff criterion using alternative clustering methods as proposed by other studies (see section Identification of Operational Taxonomic Units—OTUs). The first analyses was run in the ABGD program via a command line based using the K2P model. To maximize the potential species discovery the parameters were modified (relative value gap X = 0.1, Pmin = 0.005 and Pmax = 0.1) [31]. The BIN approach focused only on the BAST dataset in the BOLD database and the sequences from Argentina, and necessarily excluded the records from GenBank. Finally, for the GMYC analysis, ultrametric trees were generated in Beast v1.8.0 [73] using the Yule speciation and the GTR+G+I nucleotide substitution models (selected by MEGA 5 under BIC criteria), starting from a random tree, with 50 million generations, with the results being recorded every 5000 generations. The convergence of the values was checked in TRACER v1.6 [74]. The GMYC analysis [32] was implemented in the *'splits'* (SPecies' LImits by Threshold Statistics) [75] package in R, with the "single threshold" option. Only unique haplotypes were used for this analysis, given problems arising from the analysis of redundant data, as previously reported [76]. For this, the repeated identical sequences were removed using the ElimDupes tool (available at http://hcv.lanl.gov/content/sequence/ELIMDUPES/elimdupes.html).

### Identification of Operational Taxonomic Units (OTUs)

**Fig 1:**
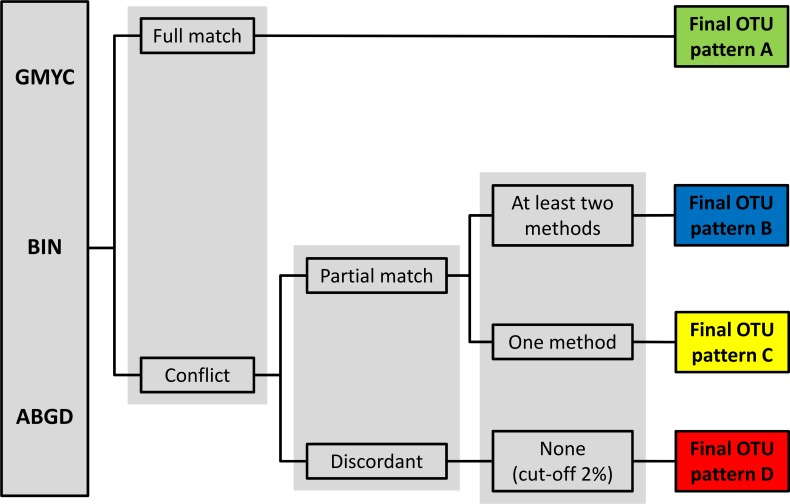
Cluster delimitation method used in this study. All clusters were first separated by 2% cutoff analysis and then tested by other delimitation methods, GMYC, BIN and ABGD. See text for further details.

Several studies propose integrative clustering delimitation methods to check for congruence in the results of the different species clustering methods into operational taxonomic units, referring to the recognition of genetic patterns within groups that supports traditional taxonomic studies [77, 78]. We used a similar approach to Costa-Silva et al. [78], where the final OTUs were classified in four categories according to the degree of correspondence with the initial NJ classification of the genetic analysis ABGD, BIN and GMYC (see Fig 1): FULL MATCH—pattern A, when all clustering methods generated the same partition (congruence between ABGD, GMYC and BIN with NJ), PARTIAL MATCH—pattern B, when two analyses generated the same cluster as NJ (GMYC and ABGD, for example), PARTIAL MATCH—pattern C, when only one method (ABGD or GMYC or BIN) generated the same cluster as NJ, or DISCORDANT—pattern D, when none of the methods are in agreement; in this case, the OTUs were delimited based on the 2% cutoff criterion of the NJ analysis as defined by the initial clustering.

## Results

A total of 1675 barcoding sequences were obtained for *Astyanax* (including published records), covering the entire area of occurrence of the genus. These sequences included 64 nominal species, 12 species identified provisionally (*A*. aff. *bimaculatus*, *A*. aff. *bockmanni* Vari & Castro 2007, *A*. aff. *intermedius* Eigenmann 1908, *A*. aff. *laticeps* Cope 1894, *A*. cf. *fasciatus*, *A*. cf. *pampa* Casciotta, Almirón & Azpelicueta, 2005, *A*. cf. *anterior* Eigenmann, 1908, *A*. cf. *asuncionensis*, Géry 1972, *A*. cf. *fasciatus*, *A*. *cf*. *jacuhiensis* Cope 1894, *A*. cf. *jequitinhonhae* Steindachner 1877, and *A*. cf. *scabripinnis*), and 40 forms identified only as *Astyanax* sp. (S1 Table). The average COI sequence size was 633 bp, with no stop codons, deletions or insertions.

**Fig 2:**
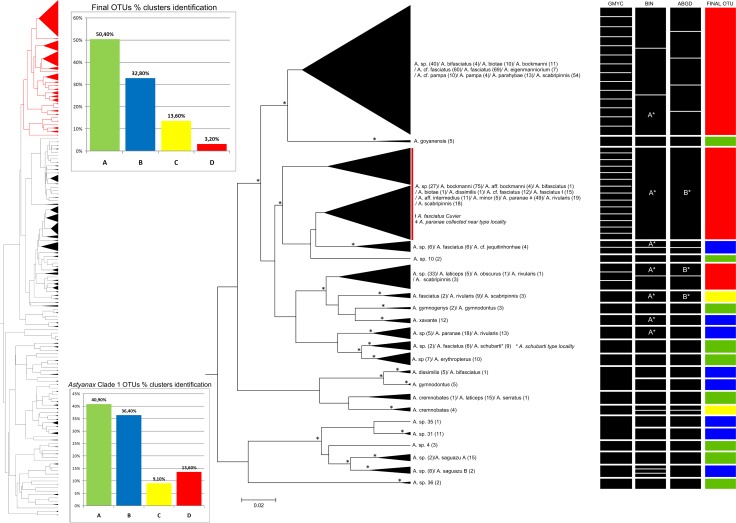
Bayesian analyses and delimitation clusters of *Astyanax* clade 1. The vertical red line indicates that the cluster *A*.*sp*.*/ A*.*bockmanni/ A*.*aff*.*bockmanni/ A*.*bifasciatus/ A*. *dissimlis/ A*. *cf*. *fasciatus/ A*. *fasciatus/ A*. *aff*. *intermedius/ A*. *minor/ A*.*paranae/ A*.*rivularis/ A*.*scabripinnis* was found at 2% cutoff NJ analysis; although separating into 2 groups, they have only 1.28% of intra-cluster genetic distance. Nodes marked with an asterisk denote probabilities greater than 0.9. BINs marked with an asterisk: groups with the same letter share the same BIN (BOLD:AAC5910). ABGD marked with an asterisk: both groups clustered together in ABGD analysis. The left tree represents the full tree, *Astyanax* clade 1 is in red. The upper histogram indicates the total number of OTUs identified in the entire data, and the lower histogram indicates the clusters identified in *Astyanax* clade 1. For further details, see S1 Table.

**Fig 3:**
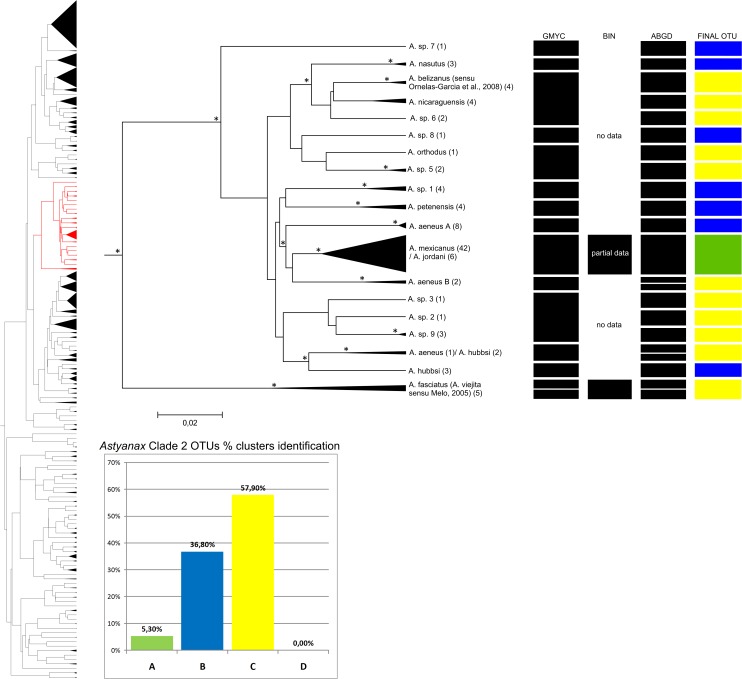
Bayesian analyses and delimitation clusters of *Astyanax* clade 2. This clade is composed by trans-Andean species. BIN results are incomplete, once that these sequences are from public databases. In the left is represented the full tree, in red is represented the *Astyanax* clade 2. Spacers internal lines inside each cluster separate and indicate the number of subgroups found in a cluster, but does not represent the proportion of individuals found in each one. Histogram indicates the proportion of clusters identification in *Astyanax* clade 2. Nodes marked with an asterisk probabilities greater than 0.9. For further details, see S1 Table.

**Fig 4:**
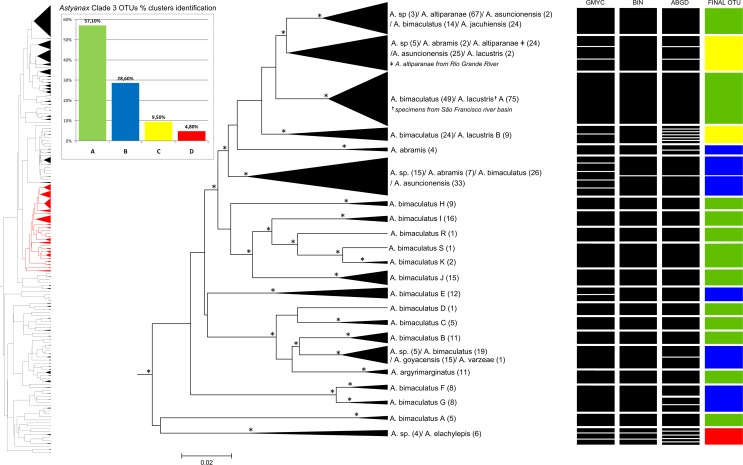
Bayesian analyses and delimitation clusters of *Astyanax* clade 3. This clade is mainly composed by *A*. *bimaculatus* species complex. In the left is represented the full tree, in red is represented the *Astyanax* clade 3. Spacers internal lines inside each cluster separate and indicate the number of subgroups found in a cluster, but does not represent the proportion of individuals found in each one. Histogram indicates the proportion of clusters identification in *Astyanax* clade 3. Nodes marked with an asterisk probabilities greater than 0.9. For further details, see S1 Table.

**Fig 5:**
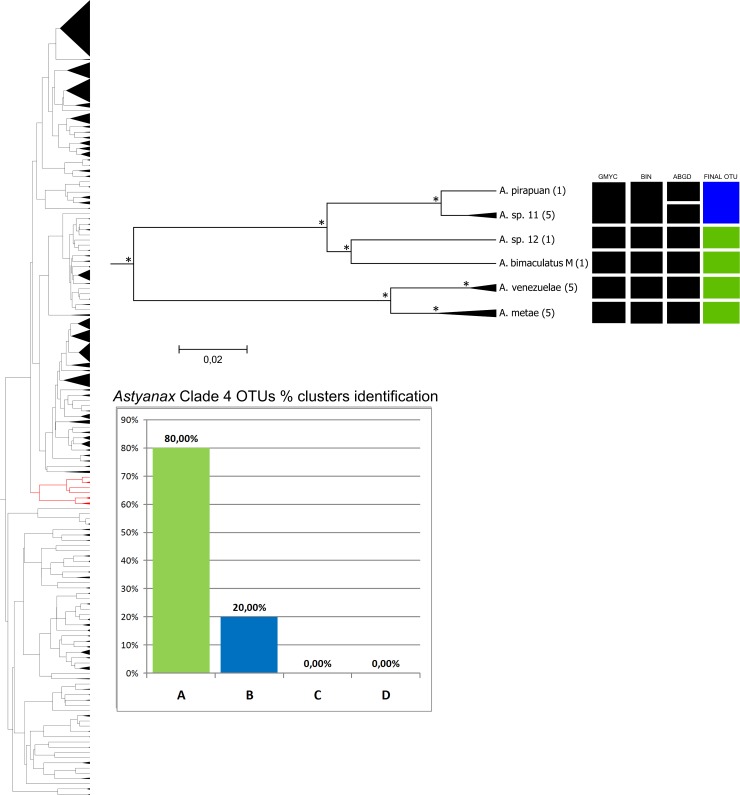
Bayesian analyses and delimitation clusters of *Astyanax* clade 4. In the left is represented the full tree, in red is represented the *Astyanax* clade 4. Spacers internal lines inside each cluster separate and indicate the number of subgroups found in a cluster, but does not represent the proportion of individuals found in each one. Histogram indicates the proportion of clusters identification in *Astyanax* clade 4. Nodes marked with an asterisk probabilities greater than 0.9. For further details, see S1 Table.

**Fig 6:**
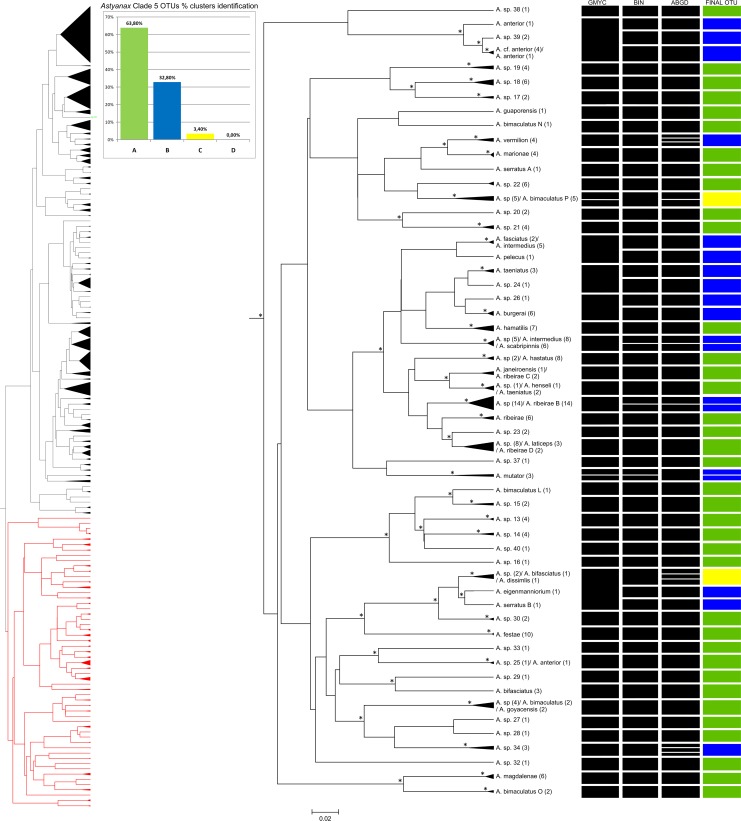
Bayesian analyses and delimitation clusters of *Astyanax* clade 5. In the left is represented the full tree, in red is represented the *Astyanax* clade 5. Spacers internal lines inside each cluster separate and indicate the number of subgroups found in a cluster, but does not represent the proportion of individuals found in each one. Histogram indicates the proportion of clusters identification in *Astyanax* clade 5. Nodes marked with an asterisk probabilities greater than 0.9. For further details, see S1 Table.

The dendrogram obtained by the NJ analysis (2% cutoff, data not shown) indicated the existence of four major (Clades 1–4) and one minor (clade 5) groups. Clade 1 includes mainly species belonging to the *A*. *fasciatus* and *A*. *scabripinnis* species complexes (Fig 2). Clade 2 encompasses the Central American species (Fig 3), and Clade 3 is formed primarily by species of the *A*. *bimaculatus* complex (Fig 4). Clades 4 (Fig 5) and 5 (Fig 6) correspond to the remaining species. This same separation was found in the bayesian analyses. Within these clades, 124 groups were identified based on NJ that correspond to potential species, with mean intragroup distances of 0.44% and intergroup distances of 18,8%, while 31 are singletons (represented by only one specimen). The final number of OTUs (congruence between methods) was 125.

The mean distance between the five major clades is 13.4%, while the mean distance within each clade ranged from 3.36% to 18.57% (Clade 2 and Clade 5, respectively; Table 1). The distances between groups of species ranged from 2% to 30.9% (S2 Table). The mean divergence within each cluster is shown in S3 Table.

**Table 1:** Mean distances within and between the five major clades.

	Clade 1	Clade 2	Clade 3	Clade 4	Clade 5
Clade 1	3.74*	1.26	1.53	1.50	1.58
Clade 2	14.42	3.36*	1.35	1.48	1.53
Clade 3	15.80	13.37	5.64*	1.54	1.57
Clade 4	15.61	15.73	16.44	7.14*	1.45
Clade 5	21.84	21.09	21.65	21.06	18.57*

K2P distance within the four major clades identified (main diagonal, marked with asterisk) and average K2P divergence between these clades (below diagonal average values and above diagonal standard error).

The analysis of the ABGD dataset using the default parameters indicated the presence of 649 species groups, which is inconsistent with our other data. However, when the intraspecific minimum distance was set to 0.4% (which is equivalent to the mean estimated intraspecific cutoff of 2%) and the value of X to 0.1, the number of groups decreased to 156 (P = 0.0040), which is clearly more consistent with the data. The BIN results are only partial, given that the GenBank sequences were not in the BOLD system, but the results of its cluster analysis were similar to those of the ABGD and the NJ analysis. The GMYC analysis indicated the presence of 149 species (confidence interval: 137–161, threshold time: -0.02811409; Table 2). The NJ approach indicated 124 putative species.

**Table 2:** Number of clusters identified on the different analysis.

	Number of clusters	Number of clusters	Number of clusters	Number of clusters
Method	NJ (Cutoff value 2%)	ABGD (relative gap width-0.1)	BIN	GMYC
All dataset	124	156	113*	149
Clade 1	22	27	23	49
Clade 2	19	22	2*	15
Clade 3	22	36	23	30
Clade 4	6	6	5	5
Clade 5	55	65	58	50

Each column shows the different method including NJ, ABGD, BIN and GMYC. Also is presented the number of clusters identified in each clade.

*Incomplete sampling (see text for details)

Species with low values of genetic interspecific distance (<2%) are found mainly in clade 1, but can also be found in other clades. A number of these clusters appear to include at least two species, indicating the occurrence of geographical regionalization (e.g., the cluster composed by *A*. *dissimilis* Garavello & Sampaio 2010 */ A*. *bifasciatus* Garavello & Sampaio 2010) or even the existence of other groups with an large distribution (e.g., cluster composed of *Astyanax* sp. */ A*. *abramis* Garavello & Sampaio 2010 */ A*. *altiparanae* Garutti & Britski 2000 */ A*. *asuncionensis* Géry 1972 */ A*. *lacustris* Lütken 1875, which includes specimens from Argentina and Brazil). Furthermore, specimens identified as *A*. *anterior*, *A*. *bifasciatus*, *A*. *bimaculatus*, *A*. *laticeps*, *A*. *scabripinnis* and *A*. *fasciatus* are present in more than one species group, indicating that they represent more than one species, with a divergence greater than 2%.

## Discussion

Following the scheme presented in Fig 1, the analyses showed that 50.4% of the OTUs correspond to pattern A, 32.8% to pattern B, 13.6% to pattern C and 3.2% to pattern D. Given the complexity of *Astyanax*, the relatively low rate of full matches of approximately 50% was not unexpected. These results in fact exceed those obtained for *Rineloricaria* (Siluriformes), another hyperdiverse fish genus, using a similar clustering approach, which gave only 41% full matches [78].

Both the NJ (data not shown) and Bayesian (GMYC) analyses revealed the presence of four major groups and one minor one. The genetic distance analyses (NJ—K2P model) confirmed the distinction of the identified clades (Table 1). Although in the NJ and GMYC we used different models (K2P and GTR+G+I, respectively) the five main clades were found in both analysis. However, the total number of species clusters identified in the NJ analyses was smaller than that observed under the other three analysis (BIN, GMYC, ABGD). In special, in the *Astyanax* clade 1 where there are specimens from two species complexes (*A*. *scabripinnis* and *A*. *fasciatus* complex) the number of clusters identified by the GMYC analyses was almost twice higher than NJ, suggesting a better capacity of this method in species separation. Some studies suggest that in complex groups the K2P model may underestimated the total number of clusters [79, 80]. Thus, we can suggest that herein GMYC, in general, was the better method for species separation. Four of these clades (1, 2, 3, and 4) were characterized by low internal genetic distances (3.74%, 3.36%, 5.64% and 7.14%, respectively), whereas clade 5 had very high levels of internal distance (18.57%). Mean intra-genus divergence in Neotropical freshwater fish is usually less than 10% (means of 8.37% in [18] and 6.8% in [19]). However, values of 10.2–12.5% were found in *Tetragonopterus* based on barcode sequences [67], although the values recorded in the present study are well beyond those found in Neotropical fish genera up to now.

On the other hand, previous studies of a small number of *Astyanax* samples found uncommonly low genetic distances between some nominal species [81]. These low divergence values were observed between closely-related species from a restricted area, such as the São Francisco basin, where values ranged from 0, between *A*. *lacustris* and *A*. *bimaculatus*, to 0.93%, between *A*. cf. *fasciatus* and *A*. *rivularis* Lütken 1875 [23]. In this case, the reduced distance between the members of the *A*. *bimaculatus* complex is consistent with their belonging to the same species (as found in the *A*. *bimaculatus / A*. *lacustris A* cluster in clade 3), although similar barcode values among members of the *A*. *fasciatus* and *A*. *scabripinnis* species complexes were also found [23]. Furthermore, DNA barcoding of specimens from Argentina indicated a reduced genetic distance (0.62%) between *A*. *eigenmanniorum* and *A*. cf. *pampa* Casciotta, Almirón & Azpelicuetta, 2005 [82].

In the present study, specimens identified as *A*. *bifasciatus*, *A*. *bimaculatus*, *A*. *laticeps* and *A*. *scabripinnis* were present in two different clades (*Astyanax* clades 1 and 5) and *A*. *fasciatus* (*Astyanax* clades 1, 2 and 5), in three clades. *Astyanax bimaculatus*, *A*. *scabripinnis* and *A*. *fasciatus* are recognized species complexes [11–14, 60]. The specimens assigned to *A*. *laticeps* are distributed over a wide area, and demand a careful review of the available evidence. *Astyanax bifasciatus* was described from the Iguaçu River basin in a review of the local *Astyanax* species [61]. In the present study, samples identified as *A*. *bifasciatus* from neighboring sites in the Iguaçu River were assigned to distinct groups, even though they cannot be distinguished from morphometric data, reinforcing the need for a systematic review of the evidence.

### *Astyanax* clade 1

This clade includes 763 individuals, 25 species and five groups identified at the genus level (*Astyanax* sp.). The different analytical approaches (Table 2) identified between 23 (BIN) and 49 (GMYC) clusters, although the NJ analysis with a 2% cutoff returned 22 groups (Fig 2). In this clade, more than one nominal species was observed in 59% of the NJ clusters.

The species of this clade are found in Brazil and neighboring western and southern countries, associated with the Paraná River basin. The large number of clusters identified and the overall genetic distance of approximately 4% impede the reliable separation of the species by DNA barcoding. In this clade, between two and 11 species were observed in the 2% threshold groups, most of which belong to the *A*. *scabripinnis* and *A*. *fasciatus* complexes. In some cases, the low levels of divergence indicate the relatively recent separation of the species or a very close relationship between them, as in the case of *A*. *paranae* and *A*. *rivularis* from the Paraná and São Francisco basins, respectively, which belong to the *A*. *scabripinnis* species complex. It is known that these two basins share their fauna [83]. It has already been reported that species from different river basins (*A*. *fasciatus*, *A*. *taeniatus* Jenyns 1842, *A*. *scabripinnis* and *A*. *intermedius*), with no intermediate forms and overlapping characters, form a “labyrinth”, which might justify a reduction in the number of species identified [7].There may be a number of potential explanations for the cases of low divergence (<2%) found in clade 1. One is the phenotypic plasticity of the species, which may hamper the reliable identification of the specimens [20]. The COI gene may also evolve at distinct rates in different groups, affecting the arrangement of the clusters [81, 84], and different groups may have distinct evolutionary histories, with some radiating more recently than others [81]. In a study of *Astyanax*, Ornelas-Garcia et al. [85] using one nuclear (RAG1) and three mitochondrial (16S, Cytb and COI) markers have identified different groups in Mesoamerica, which was attributed to a recent colonization of the region followed by rapid expansion of local populations. The invasion of Central America by ancestral *Astyanax* appears to have occurred between 3.1 and 8.1 million years ago, confirming that the radiation was relatively recent [85, 86]. An alternative explanation here is that many local populations are being described as new species due only to their restricted geographical distribution or local adaptations and should be synonymized in the future. Any one of these scenarios would demand further investigation of the status of the respective OTUs.

Specimens identified as *A*. *fasciatus* were present in three of the five clades defined in the present study. Morphological studies of the *A*. *fasciatus* complex suggest that the *A*. *fasciatus* should only be applied to specimens from the São Francisco basin, as in the original description of Cuvier [87], while other specimens identified as *A*. *fasciatus* from the Paraná basin, eastern Brazil and Central America could be assigned to other species [13]. Alternative evidence, such as cytogenetics, has indicated the existence of many different forms of *A*. *fasciatus* [88, 89], reinforcing the need for a more thorough investigation of this species complex. In the present study, clade 1 best matches the morphology and distribution of the type specimens described originally by Cuvier (Fig 2). Interestingly, the genetic distances between the specimens of this form of *A*. *fasciatus* Cuvier and others from the *A*. *scabripinnis* complex (*A*. *paranae*, collected near the type locality) are less than 1.3% (based on the analysis of 238 specimens), reinforcing the idea of a recent radiation in these fish.

In clade 1, *A*. *xavante* Garruti & Venere 2009 and *A*. *goyanensis* (Miranda Ribeiro 1944) were the only two species that could be identified unequivocally on the basis of morphology and the DNA barcode. In some cases, such as *Astyanax* sp. / *A*. *erytroptherus* Holmberg 1891 and *Astyanax* sp. / *A*. *fasciatus* / *A*. *schubarti* Britiski 1964, a redefinition of species limits (based on morphology and geographic distribution) might help solve the taxonomic problems. In many other cases, new species should be described or existing ones redescribed, through analyses including additional, faster-evolving molecular markers.

### *Astyanax* clade 2

This clade includes 100 individuals, 10 species and eight groups identified at the genus level (*Astyanax* sp.) (Fig 3). The different analytical approaches identified between 15 (GMYC) and 22 (ABGD) clusters (Table 2), while the NJ analysis with a 2% cutoff returned 19 groups and the BIN approach gave only two clusters due to a lack of data. More than one species was observed in 10.5% of the NJ clusters. This group includes all species from the west of the Andes, primarily those from Central America. The mean interspecific distance in this clade was 3.36%, with the largest distance of 10% being found between *Astyanax* sp. 8 and *A*. *fasciatus* (*A*. *viejita sensu* Melo [62]).

The cluster of *A*. *mexicanus* De Filippi 1853 specimens includes cave-dwelling fish identified as *A*. *jordani* Hubbs & Innes 1936 suggesting that they may be the same species (within-cluster distance of 0.73%). These data were derived from published records, in which were also describes marked similarities between *Astyanax* and *Bramocharax* (less than 1% divergence) and identify *Astyanax* as a polyphyletic genus [85]. Another study based on DNA barcoding also failed to separate species of the genera *Bramocharax* and *Astyanax* [17].

In this clade, a group of specimens from Lake Maracaibo in Venezuela identified as *A*. *fasciatus* represent the only sample from outside Central America, but can still be considered a species from the west of the Andes. Valenciennes described *Tetragonopterus viejita* from Lake Maracaibo [90], which was synonymized with *A*. *fasciatus* [9]. Considering the findings of the present study and those from Melo [62], it is possible to recognize *A*. *viejita* as a valid species.

In clade 2, *A*. *orthodus* Eigenmann 1907, *A*. *belizanus* Bocourt 1868 (*sensu* Ornelas-Garcia et al. [85]), *A*. *nasutus* Meek 1907, *A*. *nicaraguensis* Eigenmann & Ogle 1907, *A*. *petenensis* Günther 1864 and *A*. *fasciatus* (*A*. *vieijita*, sensu [62]) could all be identified unequivocally by the DNA barcoding.

### *Astyanax* clade 3

This clade encompasses 565 individuals, 10 species and 13 groups with most specimens identified as *A*. *bimaculatus* (Fig 4). The different approaches permitted the identification of between 23 (BIN) and 36 clusters (ABGD) (Table 2) and the NJ analysis with a 2% cutoff found 22 groups. More than one species was observed in 27.3% of the NJ clusters.

Interestingly, this clade consisted basically of species of the *A*. *bimaculatus* complex, which encompasses the *Astyanax* species in which a horizontal oval humeral spot is found, with a spot on the caudal peduncle extending to the edge of the median caudal rays [11]. Only one cluster (*Astyanax* sp. / *A*. *elachylepis* Bertaco & Lucinda 2005) and one species (*A*. *varzeae* Abilhoa & Duboc 2007) do not correspond to the external body coloration of the *A*. *bimaculatus* species group. On the other hand, other species with the same color pattern as that of the *A*. *bimaculatus* species complex (*A*. *bimaculatus* L, *A*. *bimaculatus* M, *A*. *bimaculatus* N, *A*. *bimaculatus* O and *A*. *bimaculatus* P) were assigned to clade 4 of the present study.

One cluster had four species (*A*. *altiparanae*, *A*. *jacuhiensis*, *A*. *asuncionensis* and *A*. *bimaculatus*) together with one unidentified species (*Astyanax* sp.). The mean genetic distance between the specimens in this cluster was only 0.34%, and the minimum distance of any other cluster containing specimens identified as *A*. *altiparanae* was 2.8%. The distribution of this cluster includes coastal and continental basins in the Brazilian states of São Paulo, Goiás, Mato Grosso, Minas Gerais, Paraná and Rio Grande do Sul. The existence of two clusters with specimens identified as *A*. *altiparanae* reflects recent findings in cytogenetics [91] and DNA barcoding [19]. The sum of this evidence indicates the existence of two species, treated up to now as a single taxon (*A*. *altiparanae*). The type locality of *A*. *altiparanae* is the Grande River at Volta Grande Dam in Miguelópolis, São Paulo [63], and our nearest specimen is from Colômbia, São Paulo, where the specimens were grouped with those from the group composed of *Astyanax* sp. */ A*. *abramis / A*. *altiparanae / A*. *asuncionensis / A*. *lacustris*, indicating that the specimen identified as *A*. *altiparanae* and grouped with *A*. *jacuhiensis*, *A*. *bimaculatus* and *Astyanax* sp. actually belongs to a different species. It is interesting to note that *A*. *altiparanae* is cytogenetically diverse, with 22 cytotypes described [91].

*Astyanax lacustris* from the São Francisco basin was described as *Tetragonopterus lacustris* Lütken, 1875 and this name encompasses those *Astyanax* for the forms of the *A*. *bimaculatus* complex in this basin. We identified three clusters of specimens identified as *A*. *lacustris*, with genetic distances of 2.2–2.9% between clusters. Another study notes that *A*. *lacustris* is rarely compared to *A*. *altiparanae* given the fact that the two species are found in the São Francisco and Paraná basins, respectively, even though all the traits used to differentiate these species are variable and overlapping [83]. Our results thus reinforce the need for in-depth revision of *A*. *altiparanae* that includes samples of *A*. *lacustris*.

Indeed, a recent karyotypic analysis has suggested that *A*. *altiparanae* from the Grande and *A*. *lacustris* from the São Francisco basins correspond to a single biological unit [92]. Recently a review of the *Astyanax bimaculatus* 'caudal peduncle spot' subgroup of the La Plata and São Francisco basins suggested that two nominal species–*A*. *lacustris* and *A*. *abramis*–should be considered valid [64]. In this same paper, *A*. *jacuhiensis*, *A*. *asuncionensis* and *A*. *altiparanae* are recognized as junior synonyms of *A*. *lacustris*. As our data point to three genetically differentiated groups with specimens identified as *A*. *lacustris*, further studies are clearly necessary to elucidate the systematics of this group.

In fact, one consistent group of *A*. *lacustris* from Bahia state may belong to a different species, given that it groups with *A*. *bimaculatus* from local coastal rivers, and may in fact represent a cryptic species or even *A*. *bahiensis*, recently considered to be a valid species [64]. Further studies with additional samples from the putative type locality of *A*. *bahiensis* will be needed to confirm whether one of the groups identified in the present study can be assigned to *A*. *bahiensis*. In clade 3, only *A*. *argyrimarginatus* could be identified unequivocally by DNA barcoding.

### *Astyanax* clades 4 and 5

Neither of these clades encompass clearly-defined species complexes. Clade 4 includes 18 individuals (Fig 5) and clade 5 has 229 (Fig 6), with 28 species and 28 groups of specimens identified at the genus level in the two clades (*Astyanax* sp.). The different approaches permitted the identification of between five (BIN and GMYC) and six (ABGD) clusters in clade 4, and 50 (GMYC) to 65 (ABGD) clusters in clade 5 (Table 2). The NJ analysis with a 2% cutoff identified six groups in clade 4 and 55 in clade 5. In clade 5 more than one species was observed in only 15.7% of the NJ clusters, with 65% of final OTUs with the pattern A, being one of the best resolved clades, together with the clades 3 and 4 (65% and 80% OTUs in pattern A, respectively).

While a large number of unidentified species were found in clade 5, we also identified specimens belonging to the *A*. *scabripinnis*, *A*. *bimaculatus* and *A*. *fasciatus* species complexes. These species are widely distributed in Brazil, but some species are from Colombia, Guyana and Venezuela (e.g., *A*. *metae* Eigenmann 1914, *A*. *magdalenae* Eigenmann & Henn 1916, *A*. *mutator* Eigenmann 1909 and *A*. *venezuelae* Schultz 1944) and include the only species from the west of the Andes not found in clade 2, *A*. *festae* Boulenger 1898.

While the COI gene provides good resolution at the species level, the relationships among the different groups remain unclear because of the lack of a strong phylogenetic signal [93], although some insights can be gleaned from the analyses. In particular, the marked genetic distances (up to 30.9%) found between some of the clusters identified in clade 5 and other clades reinforce the conclusion that *Astyanax* is even more complex than previously thought.

In clade 4, *A*. *pirapuan* Tagliacollo, Britzke, Silva & Benine 2011, *A*. *metae* and *A*. *venezuelae*, were identified unequivocally by the DNA barcode, as were *A*. *magdalenae*, *A*. *guaporensis* Eigenmann 1911, *A*. *festae*, *A*. *marionae* Eigenmann 1911, *A*. *vermilion* Zanata & Camelier 2009, *A*. *pelecus* Bertaco & Lucena 2006, *A*. *hamatilis* Camelier & Zanata 2014, *A*. *burgerai* Zanata & Camelier 2009, and *A*. *mutator* in clade 5. *Astyanax eigenmanniorum* was also identified by barcoding, although the other specimens of this species were included in clade 1, a similar to that found in *A*. *scabripinnis* and *A*. *fasciatus*.

## Conclusions

This study presents the most extensive investigation of *Astyanax* since the review of the last century [7]. The analysis of more than 1600 samples, including more than 70 nominal species (about half of the total number of valid species in the genus) and the other specimens identified only to the genus level, has further reinforced the complexity of the genus and the difficulty of identifying its species. We nevertheless identified five artificial clades separated by very high levels of genetic divergence (from 13.4% to 21.84%). One of the clades (clade 2) was formed by the Central American *Astyanax* forms, including *A*. *mexicanus*, the type species of the genus. We can thus speculate that this clade represents *Astyanax strictu sensu*, while the remaining groups may correspond to other genera, in line with other DNA barcoding studies of fish. This hypothesis will need to be tested with a molecular phylogeny of all the species currently included in the genus, although it was already recognized three different genera or subgenera in the *Astyanax* clade–*Astyanax strictu sensu* (which corresponds to *A*. *mexicanus* and clade 2), *Poecilurichthys* and *Zygogaster* [7].

The four major clades (1–4) presented very low intragroup genetic divergence (between 3.36% and 7.14%), whereas clade 5 presented high levels of intragroup divergence, indicating that speciation in the first four clades has been very rapid, further hampering species recognition.

Overall, only 21 morphological species (approximately 17% of the clusters in the 2% cutoff NJ analysis) could be identified unequivocally by DNA barcoding (*A*. *xavante*, *A*. *goyanensis*, *A*. *orthodus*, *A*. *belizanus sensu* Ornelas-Garcia et al., 2008, *A*. *nasutus*, *A*. *nicaraguensis*, *A*. *petenensis*, *A*. *fasciatus*, *A*. *viejita* sensu Melo, 2005, *A*. *argyrimarginatus*, *A*. *pirapuan*, *A*. *metae*, *A*. *venezuelae*, *A*. *magdalenae*, *A*. *burgerai*, *A*. *guaporensis*, *A*. *festae*, *A*. *marionae*, *A*. *vermilion*, *A*. *pelecus*, *A*. *hamatilis* and *A*. *mutator*). It is important to note, in addition, that a quarter of the diversity identified here was composed of unique specimens (31 singletons). This may be related to a combination of fast speciation, species with a broad geographic distribution, and the lack of descriptions of local morphotypes, as well as inadequate phylogenetic analyses. All these questions should be taken in account in future reviews of the genus.

## Supporting Information

S1 Table List of individuals used in this study. This list indicates the species, sampling details, vouchers numbers, clusters defined using the different approaches (NJ, GMYC, BIN and ABGD) and Genbank acession numbers.
(XLSX)

S2 Table List of pairwise between clusters distances. Pairwise distances between the clusters formed based on NJ-K2P.
(XLS)

S3 Table List of intra-cluster distances. Intra-cluster distances of the cluster formed based on NJ-K2P.
(XLS)
